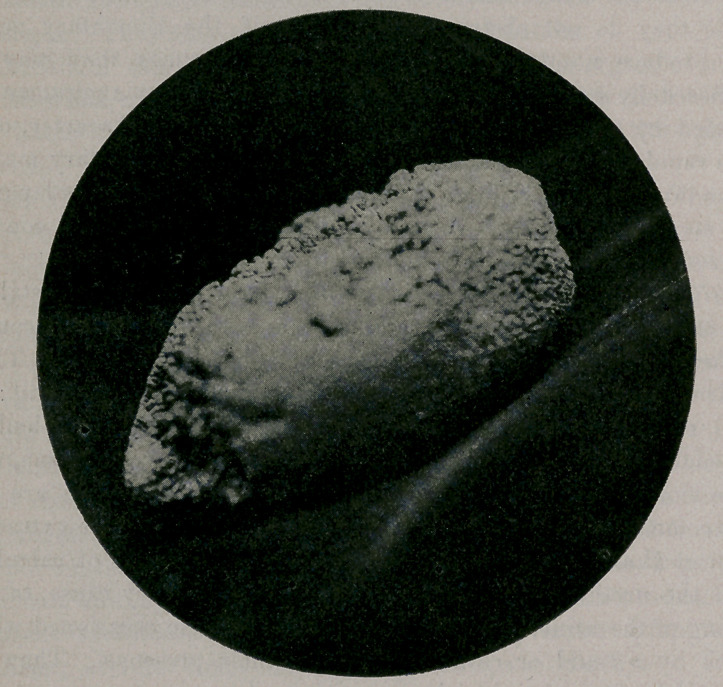# Calculi1Presented at the annual meeting of the Pennsylvania State Veterinary Medical Association, March 4 and 5, 1902.

**Published:** 1902-05

**Authors:** J. F. Butterfield

**Affiliations:** South Montrose, Pa.


### CALCULI.[1]

By J. F. Butterfield, V.S.,
SOUTH MONTROSE, PA.

Calculi are concretions which may form in every part of the animal body, but are most frequently found in the organs that act as reservoirs, and in the excretory canals. They are met with in the joints, biliary ducts, digestive passages, lachrymal, mammæ, pancreas, pineal gland, prostate, lungs, salivary, spermatic, and urinary passages. The causes which give rise to them are obscure.

Those which occur in reservoirs or ducts are supposed to be owing to the deposition of the substances which compose them from the fluid as it passes along the duct; and those which occur in the substances of an organ are regarded as the product of some nutritive irritation.

Their general effect is to irritate as extraneous bodies the parts with which they are in contact, and to produce retention of the fluid whence they have been formed.

The symptoms differ, according to the sensibility of the organ and the importance of the particular secretion whose discharge they impede.

Their solution is generally impracticable. Spontaneous expulsion or extraction by surgical aid is the only way of getting rid of them.

Arthritic calculi concretions form in joints. Similar calculi are found in the ligaments and other parts. They sometimes cause

[1] Presented at the annual meeting of the Pennsylvania State Veterinary Medical Association, March 4 and 5, 1902.

rheumatic lameness and excessive pain. They are composed of uric acid, soda, and animal matter.

Biliary calculi are most frequently found in the gall-bladder (in those animals which have one); in others, in the substance of the liver or in the branches of the ductus hepaticus. The causes of biliary calculi are also very obscure. They are usually composed of cholesterine and the yellow matter of the bile.

They may occasion violent abdominal pain. In our patients we would be unable to make a correct diagnosis. Abscesses, biliary fistulæ, and fatal effusions into the peritoneal cavity may follow.

Calculi lachrymal sometimes, but rarely, form in the lachrymal passages. They may occasion abscesses and fistulæ.

Calculi of the mammæ have been found in this organ, of a yellowish-white color, having the shape of the excretory duct. They are liable to cause abscesses, and may be removed through the abscess.

Calculi of the pancreas. These are but little known. They are supposed to resemble the salivary.

Calculi of the pineal gland. No phenomena announce their presence during life. They are composed of the phosphate of lime.

Calculi preputial are composed of sabulous and exfoliated matter. They may occasion symptoms similar to urethral and vesical calculi. Have seen them in oxen as well as in the male equine. They should be removed with the oiled finger.

Calculi of the prostate are usually composed of uric acid. Symptoms common to those of calculi of the bladder are likely to develop.

Pulmonary calculi are usually formed of carbonate of lime and animal matter. They are sometimes met with in the dead body, by butchers, and in autopsies, without seeming to have produced unpleasant symptoms during life, or they may cause symptoms of phthisis; at other times they are expelled spasmodically.

Salivary calculi are concretions usually formed of phosphate and carbonate of lime. They are developed in the substance of the salivary glands or in their excretory ducts. In the first case they may be mistaken for a simple swelling of the gland, in the second they may generally be detected by the touch. They should be extracted by incision in the interior of the mouth. If taken from the outside it would occasion a fistula that it would be difficult to close. The writer removed this specimen (weight, four ounces) from a gray mare belonging to a Mr. Vail. It was situated in Steno's duct, near the entrance to the mouth. With a mouth speculum, to hold the mouth open, and a small scalpel I was enabled to remove it readily.

Calculi of the stomach and intestines: Gastric calculi could not be formed in the stomach itself on account of the acid reaction of its contents and because of the short time the alimentary matter remains there. (In ruminants it may be otherwise.)

The anti-peristaltic movements of the intestines bring them back to the stomach from the intestines. Calculi are ordinarily formed in the large intestines; colons, rarely in the cæcum. The causes which give rise to them are ingestion of hair during shedding, or feeding ripe hairy plants, as clover, millet, soja beans, etc.; also feed rich in magnesia and lime phosphates. Intestinal concretions vary in their composition. They are light, hard, and very fetid. While they do not obstruct the passage of the alimentary mass, they produce no unpleasant symptoms. At times they may be diagnosed by examination per rectum. The violent symptoms occasioned by them are frequent colics, of a periodic character, of a more rapid course than is due to accumulation of alimentary matter in the intestinal reservoirs. Treatment: in desperate cases laparotomy may be attempted. A case we diagnosed as intestinal concretion recovered after several months without treatment.

Urinary calculi are concretions which form from the crystallizable substances in the urine, and are met with in the whole course of the urinary passages. Their causes are but little known. They receive their name from the location in which they are found: as renal, calculi of the ureters, prostate, vesical, and urethral calculi.

Renal and calculi of the ureters occasion similar symptoms, and cause violent pain at times. Urethral and vesical calculi are the most common, and are more readily diagnosed, and may be extracted by surgical aid more successfully than most other forms of calculi.

Of the urethral calculi, the obstruction which they cause to the passing of the urine, the hard tumor, and the noise occasioned when struck by a sound or catheter indicates their presence. They are removed by forceps or incisions.

Of the vesical calculi, they sometimes proceed from the kidneys. Most commonly they are formed in the bladder itself.

Frequent attempts to pass urine, sudden stoppage to its flow, and bloody urine are the chief phenomena that induce a suspicion of their existence. We cannot, however, be certain of this without an examination per rectum.

There is no such thing, probably, as a medical solvent. A surgical operation is applicable.

I will report a case of vesical calculi that I had in my practice and our mode of operating for it, original with me.

On April 18, 1900, Master Winfred Liffany, of Harford, Susquehanna county, Pa., brought to my place a white pacing gelding, very handsome, about eight years old, and weight about 1000 pounds. He was dribbling urine slightly and attempted to urinate every few minutes. He was slightly run down in condition. Master Liffany had traded for him only a few days before. Upon rectal examination I found a vesical calculi the size of a large hen's egg. This was the first, and has been the only one I have found thus far.

The boy wanted to know if I could remove it successfully. I told him I thought I could, and he left the horse with me. I deferred the operation until April 30th. In the meantime I tried, both in New York and Philadelphia, to obtain lithotomy forceps from the veterinary-instrument dealers, but was unsuccessful. I had by this time decided upon my plan of operation. With the assistance of Dr. J. G. Wilson, M.D., and Druggist Sidney Jenks, of Montrose, and my man-of-all-work, we cast the horse with the "Conkey" harness, having previously dieted him with bran mashes for twenty-four hours. I then made an entrance to the pelvic region of the abdominal cavity, the same as we do in the operation for cryptorchids, as follows: with an ordinary castrating knife I made an incision in the scrotum large enough to pass my hand. With my hand I broke down the connective tissue and fascia, and with a slight rotary movement I passed my hand right up into the inguinal region, and when I had reached about two inches above the ring I broke through the peritoneum, which brought me in the pelvic cavity near the bladder. I located the bladder at once, and seized it with the calculi and brought them out of the opening into view; held them while my assistant, Dr. Wilson, with suitable catgut, passed sutures in the muscular coat of the intending opening into the organ, leaving a loop above the point to cut through, being careful not to pass the needle through the mucous coat of the bladder.

With an ordinary scalpel we cut through the muscular portion under the loop of the catgut left for the purpose. We now shoved the muscles one side as far as we could, and cut through the mucous coat and removed the stone here shown; weight, five ounces. We now sutured the mucous coat with fine catgut, one-fourth inch apart, and drew up the sutures in the muscular portion and tied them. We now let the organ return to its normal position and allowed the horse to regain his feet. He manifested pain for a couple of hours and strained, passing a little blood.

The next morning I let him out to eat a little fresh grass and watched him for half an hour, when he urinated very naturally and did not strain at the close, as he had done when carrying the calculi. I let him out for a little exercise and to eat the fresh grass every day.

He improved rapidly. Passes a little pus at times. Sent him home, a distance of fourteen miles, May 14th, just two weeks after the operation. About four weeks from the time of the operation he strained and passed a little pus, but it lasted only two or three days, probably due to the sloughing of the sutures in the mucous coat of the bladder.

The horse was allowed to run at pasture for about three months. He gained in flesh and is in fine condition, and has been perfectly healthy since, doing all kinds of work. I see him frequently when in that vicinity.

---

Dr. Richard H. Powers was one of the successful candidates in the late examination for the United States Army, and is now veterinarian of the artillery corps about Fort Walla Walla, Washington.

## Figures and Tables

**Figure f1:**